# A Systematic Review of Non-Opioid Pain Management in Chiari Malformation (Type 1) Patients: Current Evidence and Novel Therapeutic Opportunities

**DOI:** 10.3390/jcm12093064

**Published:** 2023-04-23

**Authors:** Awinita Barpujari, Alina Kiley, Jennifer A. Ross, Erol Veznedaroglu

**Affiliations:** 1 Drexel University College of Medicine, Philadelphia, PA 19129, USA; avk53@drexel.edu; 2 Global Neurosciences Institute, Pennington, NJ 08534, USA

**Keywords:** Chiari malformation, non-opioid, opioid, pain management

## Abstract

Chiari Malformation Type I (CM) includes a range of cranial abnormalities at the junction of the skull with the spine, with common symptoms including pain and headaches. Currently, CM pain is managed medically through anti-inflammatory drugs, muscle relaxants, and opioids, while surgical management includes posterior fossa decompression. Given the adverse effects of opioid use, and an ongoing opioid epidemic, there is a need for safe, non-opioid alternatives for clinical pain management. This systematic review was performed to provide an update on the current literature pertaining to the treatment of CM pain with non-opioid alternatives. A literature search was performed in June 2022 utilizing the PubMed and Google Scholar databases, and articles were identified that included information regarding non-opioid pain management in CM patients. A total of 90 articles were obtained from this search, including 10 relevant, drug-specific studies. Two independent reviewers selected and included all relevant articles based on the chosen search criteria to minimize bias risk. Currently available treatments for neurosurgical pain management include anticonvulsants, corticosteroids, NSAIDs, anti-inflammatory drugs, NMDA receptor antagonists, local anesthetics, nerve blocks, scalp blocks, and neuromuscular blocks. While more information is needed on the use of non-opioid pain management, the present literature provides potential evidence of its efficacy amongst the CM patient population, on account of the success that non-opioid pain management has demonstrated within other neurological pain syndromes. Further research into non-pharmacological pain management would also benefit the CM population and could be generalized to related conditions.

## 1. Introduction

Chiari malformation comprises a range of cranial abnormalities at the junction of the skull with the spine, commonly affecting the posterior fossa and hindbrain [1]. It is characterized by the caudal herniation of the cerebellar tonsils below the foramen magnum and is frequently associated with syringomyelia [2]. Clinically, the presentation of CM differs amongst adult and pediatric patient populations. However, the most common presenting symptom of CM, across all patients, is pain and/or headache within the occipital and cervical region, exacerbated by routine Valsalva activities including coughing, sneezing, or laughing [3,4]. At present, it is believed that general irritation of spinal roots, particularly at the level of C1 and C2, along with irritation of nociceptors in the dura and/or blood vessels at the base of the skull, is what can lead to the pain and occipital headaches described by patients with CM. Specifically, the dorsal horn neurons that receive nociceptive information from the posterior/occipital dura are located in the C2–C4 spinal cord segments. These neurons’ cutaneous and muscle receptive fields are centered around the ears, occipital and upper neck skin, and superficial and deep neck muscles. In addition, disruption of the natural flow of cerebrospinal fluid around the herniated tonsils and the subsequent mass effect of the herniation have also been implicated as causative mechanisms of Chiari-related pain [5].

At present, Chiari malformation is managed using a combination of medical and surgical strategies. Medical therapy is used to provide symptomatic pain relief through non-steroidal anti-inflammatory drugs and muscle relaxants, whereas surgical intervention is typically implemented in patients with extreme or worsening neurological symptoms, usually through a posterior fossa decompression with or without duraplasty [6]. With a symptomatic prevalence of 1 in 1000 individuals and radiographic evidence hovering between 1 and 3.6%, CM is regarded as a common neurological disorder that demonstrates significant personal, familial, and societal burdens [7]. Despite the widespread nature of CM, there remains a significant gap in our current understanding of effective pain management amongst this population.

Managing pathological pain, such as that observed in patients with CM, remains a challenge largely because available therapeutics have limited efficacy and often cause severe adverse effects. While opioid analgesics are commonly used to manage and/or treat myriad clinical pain conditions, their use is limited due to harmful side effects including reduced GI motility, nausea, vomiting, problems with urinary retention, and increased tolerance and dependence. With long-term use, adverse effects include oversedation and increased intracranial pressure. These deleterious effects have often resulted in suboptimal pain management for neurosurgical patients [8]. Given the ongoing U.S. opioid epidemic, there is an urgent need for safe, non-opioid alternatives for the clinical pain management of CM patients.

Novel interventional and peripheral regional techniques are in development to specifically target the pathophysiological pain demonstrated by different neurosurgical patient populations, including those with Chiari Malformations. To minimize patients’ exposure to the risks of opioid use, physicians have established and implemented the use of multimodal analgesics. A multimodal regimen refers to the use of two or more medications from different classes to achieve pain relief [9]. This technique may reduce patients’ reliance on opioids, thereby decreasing the onset of opioid-induced adverse drug events. Additionally, it may also decrease the total use of perioperative opioids and may minimize costs, number of hospital visits, and length of hospital stay [10].

Presently, common non-opioid pharmacological agents for neurosurgical pain management include anticonvulsants (gabapentin, pregabalin) [11], corticosteroids (dexamethasone) [12], nonsteroidal anti-inflammatory (COX-2 inhibitors), anti-inflammatory drugs [13], NMDA receptor antagonists (methadone and ketamine) [14], local anesthetics (exparel and lidocaine) [15], nerve blocks, scalp blocks [16], and neuromuscular blocks (botulinum toxin) [17]. Of note, it is important to recognize that non-pharmacological treatments of neuropathic pain have also demonstrated significant efficacy and are also initiated at the same time as pharmacological treatment. At present, non-pharmacological treatment options for neuropathic pain include physical, surgical, and “psychocorporal” and psychotherapeutic treatment [18].

## 2. Materials and Methods

As all data came from public sources, this study was not human subject research and did not require IRB review or informed consent. We performed a search in June 2022 of the PubMed and Google Scholar databases to identify articles pertaining to non-opioid pain treatment in the Chiari Malformation patient population. For background information, the search keywords utilized included “pain treatment”, “pain management” AND “chiari” OR “chiari malformation”. For drug-specific information, the search keywords utilized included “non-opioid”, “nonopioid”, “anticonvulsant”, “antidepressants”, “local anesthetic”, “exparel”, “bupivacaine liposome”, “anticholinergic”, “botox”, “botulinum toxin”, “cannabinoid”, “cannabis”, “marijuana”, “CBD”, “THC” AND “chiari” OR “chiari malformation” with a date range between 1900 and 2022. Species was specific to humans. Concomitant conditions and/or anomalies (ex. Kimmerle anomaly, syringomyelia cysts) were allowed; however, all patients must have had a CM diagnosis. We included full-text publications that examined the use of non-opioid therapeutics for pain management amongst CM patients. Exclusion criteria included publications that were not categorized as an article, review, case report or series, case study, proceedings paper, or early access by the database criteria, and articles not published or translated into English. Case studies reporting on labor pains in CM patients were not included, as parturition pain demonstrates a confounding variable. We determined eligibility by reading the abstract of each study and eliminated those that did not satisfy the inclusion criteria. Full copies of the remaining articles were then obtained through our institution’s electronic library. Two reviewers (AB and AK) read these studies independently and determined those to include based on discussion. The same two reviewers then extracted data independently, to minimize the risk of bias, based on the predetermined study parameters and removed any duplicates. If the two reviewers disagreed, a third independent reviewer was sourced to reach a consensus about the study’s eligibility. All PRISMA guidelines pertaining to the reporting of systematic reviews were adhered to.

## 3. Results

**Figure 1:**
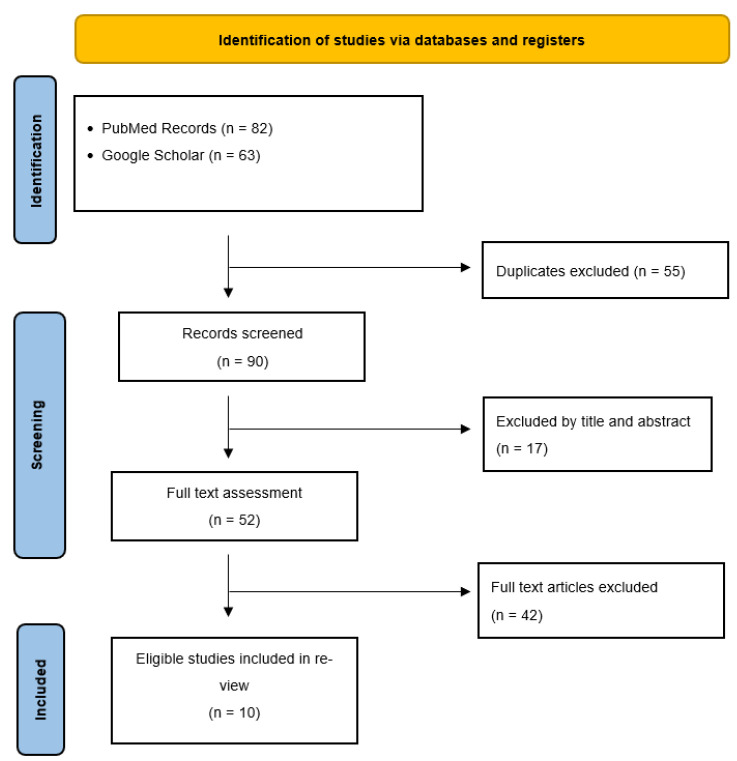
Identification of research studies via PRISMA guidelines.

**Figure 2:**
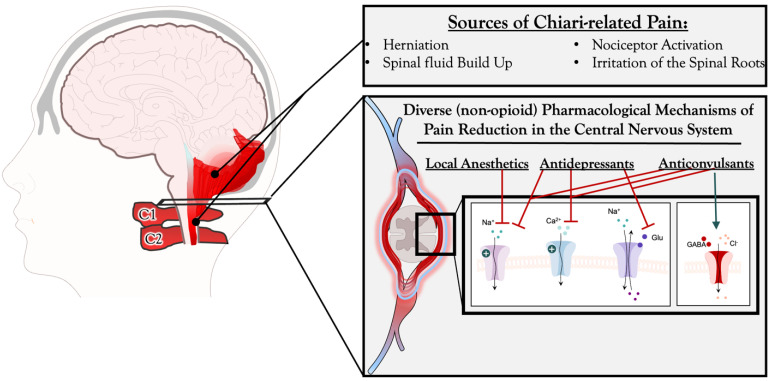
Pain and treatment schematic of Chiari Malformation Type 1 pain along with pharmacological mechanism of action for pain reduction in the Central Nervous System.

A total of 90 article results were obtained from this search. Ten relevant, drug-specific studies were identified and included (Figure 1). Articles cited in this paper had a date range of 2000–2022. A summary of the literature search is provided in Table 1. In the following sections, we provide an overview of the current literature regarding non-surgical, non-opioid, and pharmacological treatment options. As discussed previously, physicians often employ multimodal analgesics to target different components of pain. A robust table has been included to depict the various drug classes that are used to treat and/or manage CM-associated pain, along with their mechanisms of action (Table 2). These include anticonvulsants and local anesthetics for disturbed neuronal activity, antidepressants for the potentiation of descending inhibitor pathways, and analgesics to target centers involved in the development and conduction of nociceptive responses (Figure 2).

### 3.1. Anticonvulsants and Diuretics for Pain Management in Chiari Malformation Patients

Previously demonstrated pathophysiological similarities in models of epilepsy and neuropathic pain have justified the use of anticonvulsant drugs in the symptomatic management and/or treatment of neuropathic pain [19]. Vasphiades and Braswell discuss the case of a 25-year-old woman who developed headaches and visual blurring, associated with a large Chiari I malformation [20]. The patient did not report any past medical problems and was not on any medications at the time. Clinical examination showed visual acuity of 20/25 oculus uterque (OU), enlarged blind spots on the visual fields, and papilledema OU. An initial cranial MRI showed a Chiari I malformation with 12 mm of tonsillar herniation. Acetazolamide (Diamox) was initiated at 500 mg twice daily. One month following the induction of the acetazolamide therapy, a repeat cranial MRI surprisingly showed resolution of the CM. Of note, this is the only published report of Chiari I malformation associated with papilledema that has been shown to resolve with acetazolamide therapy [20]. Here, the authors suggest that patients with Chiari I malformation and pain associated with papilledema should receive a CSF flow study and be maximally treated with acetazolamide before suboccipital decompression is considered.

Vivas and colleagues provide a case series of five pediatric patients who underwent CM type I decompression with the placement of a dural graft complicated by posterior fossa hygromas and hydrocephalus that were successfully managed nonoperatively [21]. The five patients were managed nonoperatively with acetazolamide and high-dose dexamethasone; the dosages of both drugs were adjusted to the age and weight of each patient. All patients were symptom-free at follow-up and exhibited resolution of their pathology on imaging. Here, the authors conclude that de-novo hydrocephalus, in association with subdural hygromas following CM type I decompression, is rare. The reported findings indicate that such complications, following posterior fossa decompression with duraplasty can be treated non-surgically with medical management, thereby eliminating the need for CSF diversion and/or reoperation [21].

In contrast, in 1998, Demols and colleagues reported a case of intracranial hypertension (IIH) in the presence of an Arnold–Chiari malformation [22]. Here, an obese pregnant woman presented with IIH without cerebral CT scan anomaly, and despite acetazolamide treatment, her symptoms worsened and affected her neurologic state and ocular motility. In this case, a neurosurgical CSF decompression was required in order to allow for total recuperation [22]. Klocheva and colleagues examined a group of twenty-eight patients with concomitant cranio-vertebral anomalies: Kimmerle anomaly and Chiari Malformation type I [23]. Amongst these patients, the primary reasons for visiting their doctor included headache, dizziness, and sleep disturbance. The investigational drug in this case was Topamax (topiramate). One group was instructed to take 25 mg of the drug once daily (N = 16) while the other group (N = 12) was instructed to take 25 mg once a day for two weeks and then 50 mg once a day for sixty days. Here, the authors concluded that the use of topiramate resulted in reduced and/or elimination of headaches, sleep normalization, and an improvement in cerebral bioelectric activity. The drug was found to be well tolerated by these patients [23].

### 3.2. Local Anesthetics and Nerve Blocks for Pain Management in CM Patients

Prasad and colleagues have previously reported that local anesthetics and peripheral nerve blocks are commonly used for both surgical and nonsurgical analgesia [24]. Typically, non-opioid anesthetics and nerve blocks are used in conjunction with other treatments, such as NSAIDs; the combination of such treatments is termed “multimodal analgesia” and is becoming a more popular treatment strategy [9]. These techniques offer certain benefits over alternative anesthetics for particular subsets of patients and in certain specific clinical settings. These benefits include a safer and more effective method of pain relief, increased patient satisfaction, reduced hospital stay durations, and an overall decrease in healthcare costs [10].

Liposomal Bupivacaine (LB), marketed as “Exparel”, is an emerging treatment for headache pain associated with CM. Exparel is a local anesthetic used intra-operatively during CM decompression surgery and has a longer bupivacaine hydrochloride release time than standard bupivacaine [25]. The benefits of Exparel’s extended-release time include a lower risk of systemic complications, a longer period of post-surgical pain relief, and a decreased need for multiple analgesic injections. Additionally, the use of multimodal analgesia including Exparel can extend the time to the first instance of opioid use following surgery and may decrease total post-surgical opioid use [10].

A study put forth by Lu et al. in 2020 analyzed a population of pediatric CM patients who underwent decompression surgery, and either did or did not receive one dose of liposomal bupivacaine post-operatively [26]. The group that received LB and the group that did not were compared in terms of post-operative opioid use and pain control. It was found that, in the first twenty-four hours after surgery, the LB group had significantly lower opioid use (17.5 vs. 47.9 morphine mg equivalents) and lower mean pain scores on a 10-point scale (3.6 vs. 5.5) [26]. However, from twenty-four hours after surgery to discharge, there did not seem to be a meaningful difference as total opioid use and pain scores were comparable between groups. This study provides some support for the beneficial use of LB post-operatively on pain scores and opioid use twenty-four hours following surgery, but more research is needed to determine whether the benefits extend past the initial twenty-four hours [26].

A very recent study, published in May 2021 by LoPresti and colleagues, also examined the effects of liposomal bupivacaine in pediatric CM patients. The patients in this study ranged from 0–18 years old, had all undergone CM decompression surgery between January 2017 and July 2019, and either did or did not receive an intrawound injection of liposomal bupivacaine [27]. The use of intrawound LB is currently off-label for pediatric patients. The LB and no-LB groups were compared in terms of postoperative opioid consumption, pain control, length of hospital stay, and medications given at discharge. It was reported that targeted, intrawound use of LB is safe and associated with a decreased need for opioids post-operatively [27]. Additionally, LB patients did not demonstrate a need for prescriptive opioids at discharge. While this study had a small sample size, its results provide further data for the potential benefit of Exparel for pain relief in CM patients, as well as its safety in the pediatric population. This study points out the shortage of more comprehensive research on this topic and expresses a particular need for clinical trials and prospective studies.

In 2018, Levin et al. reported a case wherein a 32-year-old woman at 36 weeks gestation and a past medical history of CM type I presented with an intractable headache [28]. She was initially treated with methylprednisolone and morphine, neither of which provided any pain relief. She was then given two topical transnasal sphenopalatine ganglion blocks, obtained by applying 4% lidocaine drops into either nostril. The patient’s symptoms improved significantly, and she was discharged the same day. At the 6-month follow-up, it is noted that the patient had not had any recurrence of her headaches. Here, the authors conclude that while topical transnasal sphenopalatine ganglion block has been known for many years, it is likely understudied and underused. In this case report, the authors successfully confirm the safety and efficacy of this technique in the treatment of intractable headaches in a patient with CM type I [28].

### 3.3. Neurotoxin Therapy for Pain Management in CM Patients

Botox (Botulinum neurotoxin) represents a well-characterized and acceptable form of non-opioid pain management for chronic migraines and is an emerging therapeutic option for other types of primary headache, trigeminal neuralgia, neuropathic pain, and a number of additional pain conditions [29]. Subcutaneous injection of Botulinum toxin in patients with CM can be performed in addition to decompression surgery for further pain management, or on its own without surgery to manage CM-related head and neck pain. Until recently, severe or chronic headache pain has mostly been addressed with opioids after lifestyle modifications and anti-inflammatory drugs had failed [9]. Botulinum toxin has been shown to decrease the frequency of headaches in patients with chronic migraine disorders, and injection with Botox can increase the number of headache-free days experienced by patients [9].

Felício et al. report the case of a 31-year-old woman presenting with neurofibromatosis type I who developed right-side hemifacial spasm [30]. With magnetic resonance imaging, the authors identified a Chiari malformation type I. Botulinum toxin type A (BTX-A) injection treatments were initiated after informed consent. Five years after the BTX-A treatments were started, the patient continued to demonstrate great improvement without complaints of pain and/or any other major side effects. Here, the authors report a novel case describing the association between neurofibromatosis type I, CM type I, and hemifacial spasm and highlight the potential utility of BTX-A in providing effective pain relief [30].

These same authors, Felício et al., published a retrospective analysis evaluating five patients with CM type 1 and hemifacial spasm [31]. Three of the five patients were treated with BTX-A injections and demonstrated favorable pain-relieving outcomes. The authors concluded that hemifacial spasms may be associated with Chiari Malformation type I, particularly in younger subjects with certain phenotypic characteristics such as a short neck, and that BTX-A injection treatments may serve as an effective alternative to posterior fossa decompression [31].

### 3.4. Cannabinoid Drugs for Pain Management in CM Patients

Marijuana has been used medicinally since ancient times. While it is now known to have calming, pain-relieving, and non-addictive properties, only recently have states such as Pennsylvania and New Jersey approved the drug for medicinal use. Pain is a qualifying condition for use in both states, and many patients with Chiari Malformation use medicinal marijuana to relieve their pain symptoms. The predominant bioactive compounds in cannabis are called cannabinoids. Two types of cannabinoids are tetrahydrocannabinol (THC) and Cannabidiol (CBD). THC is known to have psychoactive properties while CBD has many medicinal benefits including anti-inflammatory and antipsychotic properties [32].

A recent publication in the *Journal of Neurological Sciences*, by Brugliera and colleagues, reported a novel case involving “therapeutic cannabis for pain management in a patient with CM type 1 during a concomitant SARS-CoV-2 infection” [2]. Here, a 32-year-old woman affected with CM type I was previously treated surgically with a midline craniectomy and C1 laminectomy and extradural filum terminale sectioning thereafter for incomplete neurological recovery. The patient reported favorable outcomes following surgery. However, in March 2020 the patient was infected with SARS-Cov-2 and presented to the emergency room with fever, tachypnea, tachycardia, cough, increases in blood inflammation indices, and hypoxia. Following admission to the hospital, the patient experienced intense suboccipital headaches and diffuse, burning paresthesiae without dermatomeric distribution in the upper limbs. The pain did not subside with the administration of non-steroidal anti-inflammatory drugs or myorelaxants. Given that opioids are contraindicated in cases of respiratory instability, the patient was prescribed oral therapeutic cannabis with an initial daily dose of 10 drops (4 mg/mL THC, 3.8 mg/mL CBD). Seven days later, the dose was increased to 15 drops daily. The patient demonstrated substantial improvement and was later discharged from the hospital. At the one-month follow-up, the patient reported significant improvement in global function and indicated that her suboccipital headaches had decreased considerably. As a result, the overseeing physicians decided to suspend cannabinoid therapy. In this case, the authors conclude that the use of cannabinoids, in tandem with an individualized rehabilitation program, may provide optimal control of occipital pain and considerable improvement in global performance in patients with CM type I and concomitant SARS-Cov-2 infection [2]. A summary of all included studies is provided in Table 3.

## 4. Discussion

Among the various craniocervical junction malformations, Chiari malformations are of particular interest given their prevalence and the intensity of their symptoms [33,34]. Given the high clinical variability and heterogeneity among CM patients, the clinical management and treatment of this condition remain a challenge. Patients can range from asymptomatic to presenting with non-specific clinical manifestations and even severe neurologic deficits. The most reported symptoms in CM patients include suboccipital headaches and severe neck pain. The described headaches are often oppressive in nature and worsen with Valsalva maneuvers (such as coughing, sneezing, or bowel movement) [33]. The reported cases of neck pain are most frequently characterized by the absence of radicular distribution and are associated with continued, burning, deep-seated discomfort in the shoulders, nape, chest, and upper limbs. Similar to suboccipital headaches, CM cases of neck pain also usually worsen with Valsalva maneuvers [33]. Other pain conditions may have similarly presenting symptoms; with an increased emphasis on research for CM pain management, patients with similar diagnoses may find great benefit for their pain management as well.

Therapeutically, there are two strategies that can be implemented to help manage CM-associated pain. The first, surgical therapy, is used to decompress the foramen magnum and is indicated for patients demonstrating intractable symptoms. The second, non-surgical therapy, is designed to specifically target and relieve the symptoms caused by neuropathic pain [33]. Of note, pain management in cases of CM remains a clinical challenge, as in any disease with low incidence in which there is a dearth of scientific evidence. Considering the extensive variability in intensity, severity, and location of CM symptoms, treatments must be personalized to each patient. At present, non-surgical pain management therapy is comprised of two types: Pharmacological and non-pharmacological.

Several methods of non-surgical therapy may often be used in conjunction with surgical therapy, either intra- or post-operatively, to reduce surgical pain and/or to minimize CM pain that was not adequately addressed through surgery. CM patients may seek further non-surgical and non-opioid intervention when surgery or other treatments have failed in alleviating their symptoms. In a 2019 study performed by Garcia and colleagues, it was noted that CM patients reported high levels of psychological symptoms, such as depression and anxiety, regardless of whether or not they had received decompression surgery [35]. While the surgical group reported lower levels of overall pain, this study demonstrated how further, evidence-based treatments such as acceptance and commitment therapy along with similar psychotherapies may be useful in further alleviating the negative side effects of CM.

In the published medical literature, there is an overall lack of robust research regarding non-opioid pain management in the Chiari Malformation patient population. By increasing this fund of knowledge, other under-researched patient populations may benefit, as the results and treatment options may be generalizable across varying pain conditions. In addition to pharmaceutical non-opioid pain management, non-pharmaceutical interventions can also play a role in the overall management and improvement of quality of life in CM patients. Data on the use of non-pharmacological interventions on pain are also lacking and may include options such as physical therapy and occupational therapy; these methods can be combined with non-opioid pharmacologic agents to provide a wide array of treatment options that can be personalized to the patient.

Limitations of our study include the heterogenous Chiari Malformation patient population and the limited number of studies that have comprehensively evaluated the use of non-opioid therapies in this niche subset of neurosurgical patients. Of note, no relevant articles regarding the use of antidepressants for pain management in CM patients were found. Case studies comprise much of the information that is currently available regarding non-opioid pain management in CM type I patients. This demonstrates a need for focused systematic clinical trials to assess the value and effectiveness of non-opioid pharmacological therapies for the management and/or treatment of pain in CM patients. In addition, the authors recognize that not all possible keywords were employed in the initial search process, which may have limited the studies that were found and included in this review. Additional keywords that may be of interest for future studies include “psychotherapy”, “alternative medicine”, “acupuncture”, and/or “homeopathic”.

## 5. Conclusions

Several evidence-based, non-opioid treatments for neurological pain management currently exist, including a number that have been tested in patients with Chiari Malformation Type I. However, there remains a major scarcity of literature pertaining to non-opioid pain treatment in CM patients. More population-specific research is needed before wider conclusions can be drawn about the efficacy of these treatments for this unique patient population. Due to the ongoing opioid epidemic, the increasing prevalence of CM [34], and the frequency and intensity of pain felt by patients, non-opioid pain management is of particular interest in this patient population. Future research may be conducted to assess the use and effectiveness of alternative pharmaceuticals, such as muscle relaxants, for CM pain management. Additionally, more research is indicated for non-pharmacological pain management techniques, such as physical and occupational therapies. Robust research in this area can increase the tools available for pain management, both for the CM population and for similar conditions. The promising results of the aforementioned treatments in other neurological pain syndromes may provide some promise for their future efficacy in CM patients, and the development of CM therapeutics may eventually be expanded and used in other craniocervical junction malformations.

## Figures and Tables

**Table 1:** Summary of database keyword searches.

Search Keywords	Hits	Included
(anticonvulsant) AND ((chiari) OR (chiari malformation))	38	4
(antidepressants) AND ((chiari) OR (chiari malformation))	7	0
(local anesthetic) AND ((chiari) OR (chiari malformation))	18	2
(exparel) AND ((chiari) OR (chiari malformation))	10	1
(bupivacaine liposome) AND ((chiari) OR (chiari malformation))	1	1
(anticholinergic) AND ((chiari) OR (chiari malformation))	6	0
((botox) OR (botulinum toxin)) AND ((chiari) OR (chiari malformation))	7	2
(cannabinoid) AND ((chiari) OR (chiari malformation))	1	0
(cannabis) AND ((chiari) OR (chiari malformation))	1	1
(marijuana) AND ((chiari) OR (chiari malformation))	1	0
(CBD) AND ((chiari) OR (chiari malformation))	0	0
(THC) AND ((chiari) OR (chiari malformation))	0	0

**Table 2:** Neuropathic pain drug classes.

Type	Mode of Action	Drug	Side Effect
Anticonvulsants	Inhibit opening neuronal voltage-dependent channels (Calcium, sodium) and GABA receptor.	Carbamazepine Gabapentin Pregabalin Topiramate	Hepatotoxicity, drowsiness, fatigue, ataxia, vertigo, gastrointestinal discomfort, headache, blurred vision
Antidepressants	Inhibit re-uptake of norepinephrine and serotonin by neurons.	Amitriptyline Duloxetine Venlafaxine	Mouth dryness, intense sedation, fatigue, diminished libido, weight loss, nausea, insomnia, headache
Local Anesthetics	Inhibit sodium influx through sodium-specific ion channels in the neuronal cell membrane.	Lidocaine Mexiletine	Dizziness, arrhythmia
Analgesics	Act through specific receptors, particularly μ receptors distributed throughout the central and peripheral nervous system blocking them.	Tramadol Dextropropoxyphene Buprenorphine Morphine Oxycodone Fentanyl Methadone	Nausea, vomiting, sweating, dizziness, mouth dryness sedation, vertigo
Diuretic (*carbonic anhydrase inhibitors*)	Works by causing an accumulation of carbonic acid by preventing its breakdown, resulting in the excretion of sodium, bicarbonate and chloride from the proximal tubule of the kidney	Acetazolamide	Fatigue, nausea, vomiting, abdominal pain, and diarrhea
Neurotoxin	Binds pre-synaptically to high-affinity recognition sites on cholinergic nerve terminals and decreases the release of acetylcholine	Botulinum neurotoxin	Muscle paralysis, headaches, flu-like symptoms, and allergic reactions
Cannabinoids	Binds and activates two types of G-protein-coupled receptors, CB1 and CB2 which results in an inhibition of the release of the neurotransmitters acetylcholine and glutamate while indirectly affecting y-aminobutyric acid, N-methyl-D-aspartate, opioid and serotonin receptors.	Cannabis	Altered senses, changes in mood, difficulty with cognition, hallucinations, delusions and impaired memory.

**Table 3:** Summary of articles included in review.

Name of Study	Journal	Author	Year	Number of Patients	Sex of Patients	Age	Therapy Specifics	Conclusions
Therapeutic cannabis for pain management in a patient with chiari malformation type i during concomitant SARS-CoV-2 infection	Journal of Neurosurgical Science	Luigia Brugliera et al. [2]	2023	1	F	18+	pharmacological	Support for cannabis in improvement of CM headaches
Resolution of Chiari I malformation following acetazolamide therapy	Seminars in Ophthalmology	Michael Vaphides et al. [20]	2007	1	F	18+	pharmacological	CM resolution, supporting CSF flow study for CM patients
Management of hydrocephalus and subdural hygromas in pediatric patients after decompression of Chiari malformation type I: case series and review of the literature	JNS Pediatrics	Andrew C. Vivas et al. [21]	2018	5	M, F	<18	surgical, then pharmacological	Supporting non-operative management of de novo hydrocephalus following CM decompression
Association of idiopathic intracranial hypertension- Arnold-Chiari deformity- danger!	Bull Soc Belge Ophtalmol	P Demols et al. [22]	1998	1	F	18+	surgical	supporting link between IIH and CM
The use of topiramate at patients with combined craniovertebral anomaly	Zh Nevrol Psikhiatr Im S S Korsakova	E G Klocheva et al. [23]	2019	28	M, F	18+	pharmacological	supporting use of Topamax for reduction of headache in CM
Effects of intraoperative liposomal bupivacaine on pain control and opioid use after pediatric Chiari I malformation surgery: an initial experience	JNS Pediatrics	Victor M Lu et al. [26]	2020	18	M, F	<18	pharmacological	supporting intraoperative LB use to reduce pain scores within 24 h post-op
Intrawound Liposomal Bupivacaine in Pediatric Chiari Decompression: A Retrospective Study	Pediatr Qual Saf	Melissa A LoPresti et al. [27]	2021	30	M, F	<18	pharmacological	supporting LB use for decreased opioid use and pain following CM decompression
Sphenopalatine Ganglion Block Successfully Treats Migraines in a Type 1 Arnold Chiari Malformation Pregnant Patient: A Case Report	A&A Practice	Danielle Levin et al. [28]	2018	1	F	18+	pharmacological	supporting transnasal sphenopalatine ganglion blocks in resolution of CM headaches
Hemifacial spasm in a patient with neurofibromatosis and Arnold-Chiari malformation: a unique case association	Arq Neuropsiquiatr	Andre Carvalho Felicio et al. [30]	2007	1	F	18+	pharmacological	supporting botulinum toxin for HFS in CM
Young onset Hemifacial Spasm in patients with Chiari type I malformation	Parkinsonism Relat Disord	Andre Carvalho Felicio et al. [31]	2008	5	M, F	18+	pharmacological	supporting botulinum toxin for HFS in CM

## Data Availability

No new data were created.
